# 小细胞肺癌的的分子发病机制

**DOI:** 10.3779/j.issn.1009-3419.2010.11.18

**Published:** 2010-11-20

**Authors:** Sandra P. D'ANGELO, M. Catherine PIETANZA, 红洁 李, 晓平 李

**Affiliations:** 1 Department of Medicine; Thoracic Oncology Service; Division of Solid Tumor Oncology; Memorial Sloan-Kettering Cancer Center and the Weill Medical College of Cornell University; New York, NY USA; 2 天津市第一中心医院ICU科; 3 天津市第一中心医院胸外科

**Keywords:** SCLC, 染色体, 抑癌基因, 癌基因, 酪氨酸受体激酶, 进化通路

在美国，SCLC（small cell lung cancer, SCLC）占所有确诊肺癌病例的13%。尽管其对放、化疗均较敏感，但SCLC复发迅速，患者的五年生存率仅为5%。这种极差的预后可能要归咎于治疗方式的进展缓慢，因为在过去三十年中，针对SCLC的医疗及护理方案并无明显变化。SCLC具有独特的生物学特征，伴随着特定的分子和细胞改变，这也是现今的研究热点。这里，我们总结了可能导致SCLC发病机制的改变；染色体的变化；抑癌基因、癌基因以及信号通路的失调；受体酪氨酸激酶、生长因子和细胞标志物的上调；以及进化通路的持续存在。以上各项也是治疗的潜在靶点，许多生物学制剂亦在研发中。

## 前言

在美国，每年有3万患者被确诊为SCLC。SCLC具有独特的自然病程，它倍增时间较短，生长分数高、早期广泛转移以及对放、化疗具有独特的初始反应。SCLC的一线治疗包括：应用顺铂或卡铂+依托泊苷的联合化疗（局限期SCLC加用放疗），应用上述治疗方案可使总有效率达到60%-80%。但是，所有进展期及大部分局限期SCLC患者都会在初始治疗完成后数月内复发。对初始治疗保持一定程度的反应达3个月或以上的患者被认为是“敏感的”。这些患者对于二线化疗有反应的可能性更高，尽管疗效仅能达到一线治疗的一半，患者生存期平均仅有6个月左右。“难治性”患者或对初始治疗无反应，或在完成治疗后的3个月内出现疾病进展。“难治性”患者，对二线化疗的有效率低于10%，生存期仅为3个月至4个月^[1]^。

由于复发性SCLC患者的预后不佳，探索一种新的治疗策略显然是必要的。针对分子和细胞异常的研究可能会有效。在该综述中，我们将聚焦于染色体改变、抑癌基因、癌基因、异常信号通路、受体酪氨酸激酶和生长因子、细胞标记物、以及已知的SCLC的进化通路等。我们亦会重点介绍临床前和临床上针对这些过程的正在研发中的生物制剂。

## 染色体异常

**染色体改变**。在SCLC和其它上皮肿瘤中均可见多种染色体异常，从而反映出基因的不稳定性^[2]^。大多数的SCLC存在影响多个染色体位点的基因缺失，缺失经常发生于3p、5q、13q以及17p上，这些位点是包括p53在内的一系列抑癌基因的基因座。比较基因组杂交分析显示：大量SCLC在1p、2p、3q、5p、8q和19p染色体中存在基因扩增。这些区域编码为我们所熟知的癌基因，如MYC和KRAS。在SCLC细胞系中可见1p、2p和3q的扩增及18q的缺失，显示出该疾病具有更高的侵袭性^[2]^。

在超过90%的SCLC中可见染色体3p上等位基因的缺失，而这被认为是肺癌的一个早期事件^[3]^。已被鉴定出的包含缺失的明显区域包括3p21.3、3p12、3p14.2以及3p24^[4]^。这些区域的数个基因具有肿瘤抑制活性，而通过表观遗传学机制常常会表达缺失。

3p21上的抑癌基因包括RASSF1A、FUS1、SEMA3B和SEMA3F。RASSF1A基因可被肿瘤获得性启动子超甲基化灭活^[5]^。它编码类似于R A S效应蛋白的一种蛋白质，且在超过90%的SCLC中失活^[5]^。RASSF1基因参与细胞周期通路、细胞凋亡以及微管的稳定性^[6]^。而在所有的SCLC中FUS1基因均失去了蛋白表达能力^[7]^。野生型FUS1具有G_1_期阻滞以及细胞凋亡的能力^[3]^。一项在晚期肺癌中正在进行的临床试验是通过FUS1-纳米颗粒介导的运输完成的^[7]^。

脆性组氨酸三联体（FHIT），位于3p14.2，可发生同型缺失且发生与100%的SCLC中^[8]^。FHIT调节死亡受体基因。临床前研究表明将野生型FHIT转染至肺癌细胞中可诱导凋亡^[9]^。3p24区域包含RARβ基因，其在72%的SCLC中呈甲基化状态，该状态导致其表达缺失。RARβ在上皮细胞的生长调节以及肿瘤生成抑制中发挥重要作用^[10]^。

**端粒酶**。端粒是位于染色体末端的核苷酸重复序列（TTAGGG），可保护染色体以免其降解和细胞死亡。每次细胞分裂均可导致DNA序列中的端粒缺失。端粒酶是一种RNA依赖性的DNA聚合酶，通过在复制细胞中合成端粒重复序列以弥补DNA序列^[11]^的缺失。端粒酶由一个催化亚基（hTERT）和一个端粒酶RNA亚基（hTR）组成。

在终末分化细胞中，端粒酶活性处于沉默状态。细胞通过复活端粒酶以补偿端粒重复序列的丢失而获得永生^[12]^，从而导致无限制的细胞分裂。超过98%的SCLC存在hTR与端粒酶活性的上调^[11, 13]^。靶向作用于端粒酶的一系列制剂正处于早期临床试验阶段，包括GV1001（合成肽疫苗，hTERT）、端粒溶解素（OBP-301，端粒酶特异性溶瘤病毒）和GRN163L（hTR RNA模板区域的拮抗剂）^[14, 15]^。

## 抑癌基因

**p53**。抑癌基因p53，位于染色体17p13.1，是细胞的“看门人”，通过调节细胞存活和损伤反应通路以保护细胞免疫的遗传不稳定性作用。它通过靶向作用于涉及细胞周期停滞期（G_1_和G_2_）[p21基因]（见图 1）、DNA修复[GADD45]和凋亡[BAX]（见表1和2）的下游基因而作为细胞增殖的一种负性调节因子^[8]^。

**1:**
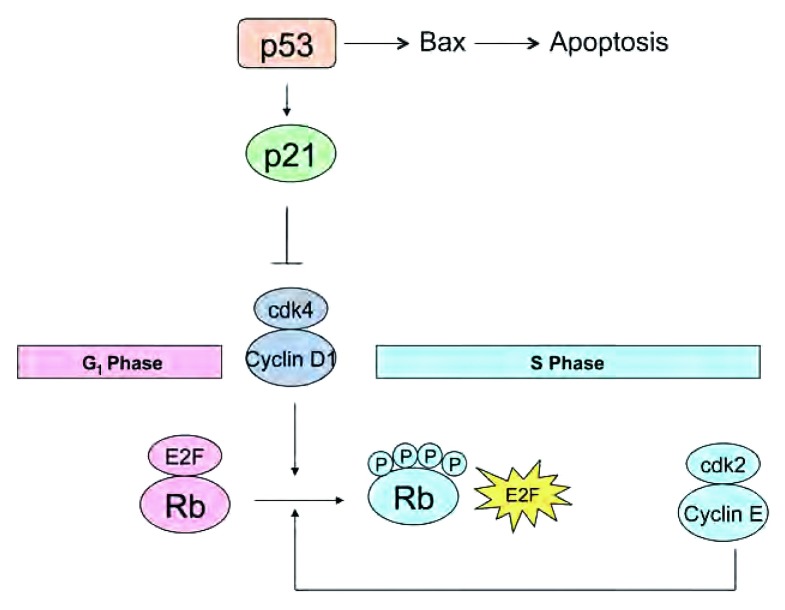
细胞的“看门人”，p53作为细胞增殖的负性调节因子而发挥作用。P通过靶向作用于与cyclin D1/cdk4复合物结合的p21，p53可使细胞停滞于G_1_期。p53诱导凋亡的其中一种机制便是通过增加Bax的合成；随后Bax阻滞具有抗凋亡作用的Bcl-2（见图 2）。p16/Rb通路介导G_1_/S期转化。Cyclin D1/cdk4复合物可磷酸化Rb。当Rb为磷酸化状态时，E2F被激活，可使细胞转化至S期。在S期，cyclinE和cdk2可调控Rb的磷酸化。 As the gatekeeper of the cell, p53 acts as a negative regulator of cellular proliferation. By targeting p21, which binds to the Cyclin D1/cdk4 complex, p53 causes cell cycle arrest in G_1_. One mechanism by which p53 causes apoptosis is through the increased synthesis of Bax; Bax then blocks Bcl-2, which is anti-apoptotic (see also Fig. 2). The p16/Rb pathway mediates G_1_/S phase transition. The Cyclin D1/cdk4 complex phosphorylates Rb. When Rb is phosphorylated, E2F is active, causing transition to S phase. During S phase, cyclin E and cdk2 control the phosphorylation of Rb.

p53在SCLC的发展过程中发挥主要作用^[8]^。大约在90%的SCLC中存在p53失活性突变，其中多数为DNA结合区域的错义突变，少数为纯合子缺失^[3]^。在40%-70%的SCLC中存在异常的p53蛋白表达^[8]^。p53突变与吸烟相关；特别是GC与TA的易位由烟草中的苯芘致癌物所导致^[4]^。

在癌细胞中突变型p53具有较高的表达及较长的半衰期，从而使其适用于肿瘤的免疫疗法。临床前研究显示包含重组腺病毒（DC-Ad-p53）的人野生型p53转染至树突状细胞后，由该树突状细胞组成的疫苗可引发抗肿瘤反应^[16]^。这些发现使在广泛期SCLC患者中应用这种疫苗的治疗进入了Ⅰ/Ⅱ期临床试验阶段，这些患者均已完成了以铂类为基础的一线化疗^[17]^。相关研究人员表示这种疫苗是安全的，且在少数患者中可达到部分缓解，如结合补救性化疗，可提高客观疗效^[17]^。在SCLC患者中使用疫苗与化疗联合治疗的随机Ⅱ期临床试验正在进行中（www.clinicaltrials.gov）。

**视网膜母细胞瘤**。P16INK4-cyclin D1-CDK4-RB通路主要介导细胞周期中G_1_/S期的转化^[18]^，在SCLC中，最常见的通路异常发生于RB基因^[18]^。低磷酸化RB是其生长抑制状态，可调控G_1_/S期转化所必须的转录因子E2F1、E2F2及E2F3^[18]^。当与低磷酸化的RB结合时，E2F处于失活状态，可使细胞停滞于G_1_期。cyclinD1/CDK4复合物可使RB发生磷酸化，随后可释放E2F，E2F恢复活性并使细胞转化至S期^[4]^（见图 1）。此外，磷酸化的RB可通过抑制其它促凋亡靶基因以抑制凋亡，这些基因包括凋亡蛋白酶激活因子-1（Apaf-1）和半胱氨酸蛋白酶^[19]^。在S期，cyclinE和cdk2调控RB的磷酸化^[4]^。

RB基因可发生的突变类型包括缺失、无义突变和剪切异常^[18]^。在超过90%的SCLC患者可见Rb的完全缺失或者突变^[20]^。由于所有正常细胞均可表达功能性Rb，因此靶向作用于具有失活或缺失Rb细胞的药物可能是SCLC患者治疗的恰当候选。这些药物包括热休克蛋白90（Hsp90）抑制剂，将在下文中进入深入讨论。

## 无受体癌基因

**Bcl-2基因**。Bcl-2是可以调控细胞死亡以及诸如凋亡、坏死和自噬等机制的蛋白质家族成员之一^[21-23]^。它位于线粒体和内质网中^[24]^。Bcl-2家族由抗凋亡蛋白（bcl-2、bcl-x和Mcl-x）和促凋亡蛋白（ba x、ba k和bad）组成。当肿瘤坏死因子受体凋亡诱导配体（tumor necrosis factorreceptor apoptosis-inducing ligand, TR A IL）与死亡受体（DR4或DR 5）结合的外源性信号或DNA损伤剂诱导的内源性信号可引发程序性细胞死亡。促凋亡蛋白和抗凋亡分子之间的平衡调节细胞色素c从线粒体的释放，由此调控半胱氨酸蛋白酶激活和细胞死亡的比率^[25]^。Bcl-2通过抑制bax及bak的促凋亡作用以促进细胞存活（见图 2）。

**2:**
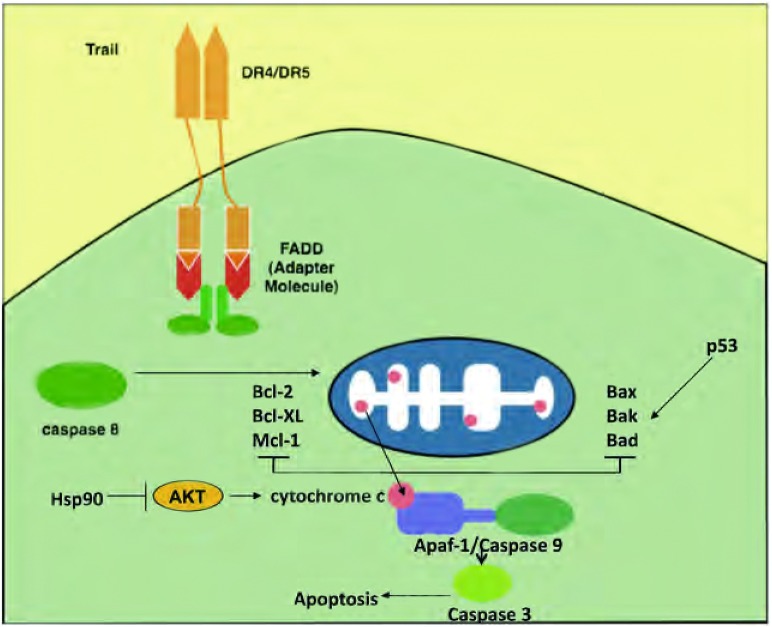
TRAIL（肿瘤坏死因子凋亡诱导配体）与死亡受体（DR4/5）结合的外源性信号或DNA损伤剂有点哦啊的内源性信号可引发程序性细胞死亡。促凋亡蛋白（Bax、bak和bab）与抗凋亡蛋白（bcl-2、bcl-xl、mcl-1）之间的平衡调控线粒体中细胞色素c的释放，这将最终导致半胱氨酸蛋白酶的活化以及细胞死亡。Hsp90可对宿主蛋白进行蛋白质折叠，这些蛋白包括：AKT、MET、bcl-2、端粒酶及Apaf-1。Hsp90所致的AKT失活可使细胞色素C活化，随后导致细胞凋亡（引自Haura等^[25]^）。 Programmed cell death occurs through extrinsic signaling, when TRAIL (tumor necrosis factor apoptosis induced ligand) binds to the death receptors (DR4/5), or through intrinsic signaling induced by DNA-damaging agents. The balance between pro-apoptotic proteins (Bax, bak and bad) and anti-apoptotic proteins (bcl-2, bcl-xl, mcl-1) regulates the release of cytochrome c from the mitochondria which ultimately leads to caspase activation and cell death. Hsp90 is responsible for protein folding of client proteins such as AKT, MET, bcl-2, telomerase and Apaf-1. AKT inactivation by Hsp90 leads to activation of cytochrome c which subsequently leads to apoptosis. Adopted from Haura, *et al* ^[25]^.

bcl-2的表达变化可导致人类恶性肿瘤的发病及进展^[26]^。抗凋亡bcl-2蛋白在许多恶性肿瘤中过表达，而它们的表达通常与药物敏感性相关^[27, 28]^。75%-95%的SCLC中存在Bcl-2的上调^[29]^。在SCLC细胞系及异种移植物模型中抑制bcl-2呈现抗肿瘤活性^[30-32]^。oblimerson为一种早期临床研发药物，是一种靶向作用于bcl-2的反义寡核苷酸，在癌症和白血病B组（Cancer and Leukemia Group B）实施的一项Ⅱ期试验中，oblimerson联合足叶乙甙、卡铂的方案并未改善患者的生存^[33]^。AT-101、BT263和X15-070070是新型制剂，通过与活化疏水口袋的结合而抑制bcl-2和其它家族成员，这些药物均处于SCLC患者的临床试验中。

**Myc基因**。Myc基因家族编码核DNA结合蛋白、c-MYC、N-MYC以及L-MYC，作为转录因子发挥作用从而调节细胞增殖、凋亡及分化^[34-36]^。蛋白的过表达与基因扩增或转录失调所导致的MYC激活较常见^[37]^。据报道18%-31%的SCLC中可见MYC的激活^[38]^，且与生存期的缩短有关^[4]^。

## 信号通路

**磷酸次黄嘌呤核苷酸3-激酶/AKT/mTOR途径**。磷酸次黄嘌呤核苷酸3-激酶（PI3Ks）为脂蛋白激酶家族，调节诸如细胞增殖、存活、运动、粘附及分化等细胞功能^[4]^。由酪氨酸激酶受体及G蛋白偶联受体激活后，PI3Ks可通过生成磷脂而使源自多种生长因子及细胞因子的信号转化为细胞内信息，从而激活下游的效应途径，包括AKT以及丝氨酸/苏氨酸蛋白激酶^[4, 39, 40]^。PI3K/AKT通路的主要下游介导因子为mTOR，它的作用靶点是核糖体蛋白S6激酶1（S6K1）和真核翻译起始因子4E结合蛋白1（4EBP1）^[41]^，可调节蛋白合成^[39, 40]^。PTEN为肿瘤抑制因子，是最重要的PI3K信号通路的负性调控因子^[39, 40, 42]^。

在SCLC中PI3K/AKT/mTOR信号通路是有缺陷的：SCLC细胞具有组成性活化PI3K^[43]^以及隐匿性PI3K和PTEN突变^[44]^；在70%的SCLC患者中可见AKT的磷酸化^[45]^；与Ⅱ型上皮细胞相比，SCLC中mTOR、S6K1和磷酸化4EBP1的蛋白表达均有所增加^[46]^，这些变化导致了SCLC的生长、生存以及化疗耐药。应用特异性PI3K抑制剂渥曼青霉素（wortmannin）、LYS294002及处理SCLC细胞，可减缓细胞生长并诱导细胞凋亡^[43]^。体外及体内研究均显示应用雷帕霉素衍生物RAD001拮抗mTOR可显著抑制SCLC的肿瘤生长^[46]^。同时应用LYS294002^[44]^和RAD001^[46]^可增强足叶乙甙的促凋亡作用。

PI3K/AKT/mTOR通路抑制剂正处于早期临床试验阶段。一项随机Ⅱ期研究显示，对完成标准一线化疗的87例广泛期SCLC患者应用双倍剂量的西罗莫司脂化物（temsirolimus, CCI-779）治疗并未改善预后^[47]^。依维莫司（RAD001）单药治疗40例曾接受过治疗的SCLC患者，耐受性良好，但在非指定人群中疗效有限^[48]^。一项评价顺铂、依托泊苷及依维莫司对未曾接受过治疗的广泛期SCLC患者的疗效的Ib期试验正在进行中（ww.clinicaltrials.gov）。多种PI3K抑制剂（如：XL147、CAL-101、PK-866、GDC-0941、BKM-120）、PI3K和mTOR的双重抑制剂（如：BEZ235、BGT226、XL765、SF1126、GSK1059615）、AKT抑制剂（如：哌立福辛、VQD002、MK2206）和mTOR抑制剂（如：AP23573、AZD8055、OSI1027）均已进入早期临床试验阶段^[39, 40]^。

## 受体酪氨酸激酶和生长因子

在SCLC，数个受体酪氨酸激酶呈过表达。受体酪氨酸激酶通过活性氧的改变及下游信号转导分子的激活而参与细胞增殖、迁移和存活（见图 3）^[4]^。受体酪氨酸激酶抑制剂已成为SCLC的潜在的抗肿瘤药物。

**3:**
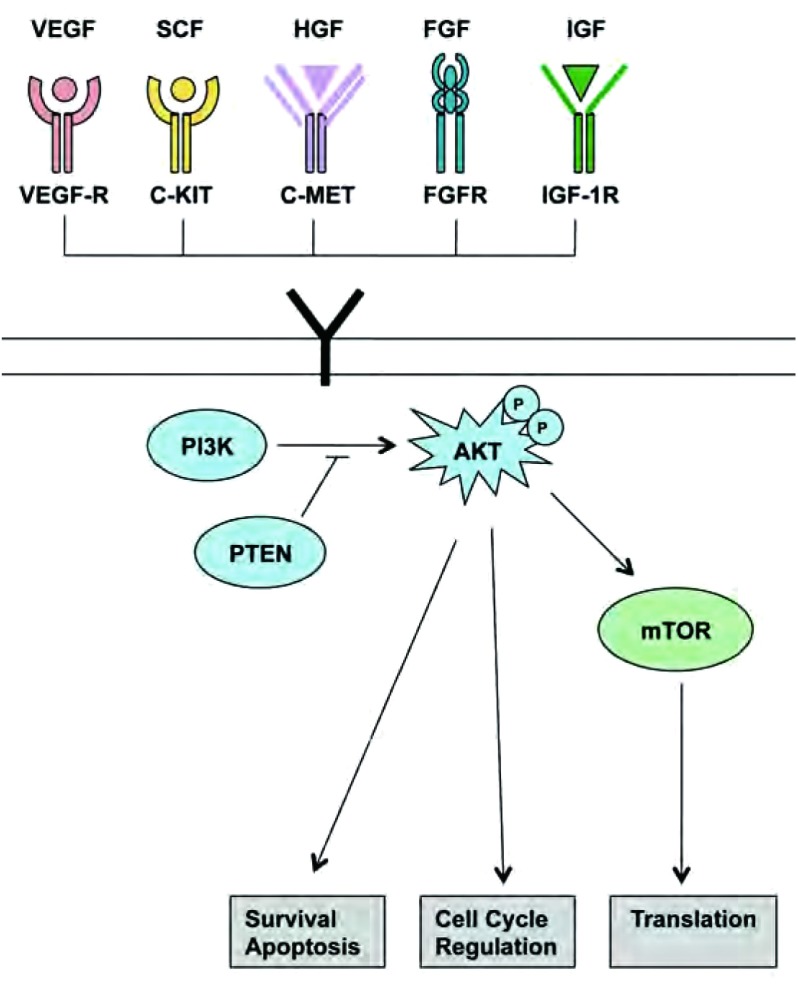
在SCLC中，受体酪氨酸激酶及其配体通常呈过表达状态。缺陷性受体酪氨酸激酶及它们各自的生长因子包括：VEGFR/VEGF, c-Kit/SCF, c-MET/HGF, FGFR/FGF以及IGF-1R/IGF。通过多种通路的激活，包括在SCLC中失调的PI3K/AKT/mTOR通路，在存活、凋亡、细胞周期调节和翻译过程中发挥作用。 Receptor tyrosine kinases and their known ligands are often overexpressed in SCLC. Defective receptor tyrosine kinases and their respective growth factors in SCLC include VEGFR/VEGF, c-Kit/ SCF, c-MET/HGF, FGFR/FGF and IGF-1R/IGF. Through activation of multiple pathways, including the PI3K/AKT/mTOR pathway that also is known to be dysregulated in SCLC, they are involved in survival, apoptosis, cell cycle regulation and translation.

**c-Kit**。c-Kit是PDGF/c-Kit受体酪氨酸激酶家族的成员。与其配体干细胞因子（SCF）结合后，通过激活JAK – STAT、PI3K和MAP激酶通路可启动细胞生长和分化^[45]^，从而促使SCLC的发病^[49]^。通过SCF/c-Kit受体的自分泌环以及内源性SCF敏感性的增加，细胞生长可被增强^[50]^。在79%-88%的SCLC细胞系中可见c-Kit的表发，而在57%-76%的SCLC中可见c-kit和SCF的同时表达^[50]^。

研究表明酪氨酸激酶抑制剂伊马替尼通过使c-KIT的失活可抑制SCLC细胞的生长^[51]^。然而，与临床前数据相比，伊马替尼在SCLC中并未显示出在各种Ⅱ期临床试验中所证实的疗效：未经治疗或复发的病例^[52]^，与伊立替康和卡铂联合作为一线治疗^[53]^，作为伊立替康、顺铂一线治疗后的维持治疗^[54]^，c-kit表达上调的再发性SCLC^[55, 56]^。

**c-Met**。c-Met受体酪氨酸激酶可被其配体、肝细胞生长因子/离散因子（HGF/SF）、下游信号分子如Grb2（生长因子受体蛋白2）、PI3K的P85亚基、STAT3和Gab1（Grb2结合蛋白-1）所激活^[4]^。这一信号通路的激活可引发细胞增殖、存活、运动、浸润至细胞外基质及微管形成^[4]^。

研究证实SCLC中可见c-Met的过表达和扩增，且高水平的HGF与预后不良有关^[4, 45]^。HGF/c-Met使SCLC更具侵袭性，调节c-Met/HGF通路可改变细胞的运动及迁移能力^[57, 58]^。在SCLC中，应用siRNA抑制或SU11274（c-Met小分子抑制剂）阻断c-Met可使下游信号转导因子的活性降低^[59]^。多种C-MET抑制剂，如XL880、ARQ197、MK2461和PF2341066，现正处于早期临床试验阶段。

**胰岛素样生长因子-1受体**。胰岛素样生长因子受体（IGF-1R），是受体酪氨酸激酶胰岛素受体亚类的成员，可被其配体IGF-1、IGF-2以及信号有丝分裂、抗凋亡和转化活性激活^[45]^。在SCLC细胞系中IGF-1R及其配体IGF-1的表达水平升高。超过95%的SCLCs的IGF-1蛋白水平升高^[4, 45, 60]^。IGF-1R可激活SCLC中的PI3K-AKT通路而在疾病的发生、生长以及化疗耐药中发挥作用^[45]^。

使用单克隆抗体（包括IMC-A12和AMG479）及受体酪氨酸激酶抑制剂（如NVP-ADW742，BMS-554417和AG_1_024）可抑制IGF-1R通路，其中单克隆抗体已进入临床试验阶段，受体酪氨酸激酶抑制剂仍处于临床前研究阶段^[61]^。在SCLC细胞系中，IMC-A12可在抑制PI3K-AKT信号的同时抑引起生长抑制和化疗增敏^[62]^。单独使用NVP-ADW742具有较强的抗肿瘤活性，而分别与IGFR-1和c-kit抑制剂AG_1_024和AG_1_296联用时，对SCLC的增殖、抑制和凋亡诱导具有协同作用^[63, 64]^。

**成纤维细胞生长因子受体**。酪氨酸激酶成纤维细胞生长因子受体家族共有四个不同的亚型（FGFR1-4）。成纤维细胞生长因子（FGFs）与FGFR结合后，受体可与许多信号蛋白相互作用并激活Ras/Raf/MEK/Erk1, 2及PI3K-AKT信号通路^[65]^。

碱性成纤维细胞生长因子（bFGF或FGF-2）在SCLC中具有生物学作用^[45]^。SCLC患者血清中FGF-2的水平升高与预后不良及血管生成增强具有相关性^[66]^。FGF-2可刺激SCLC的生长并导致化疗药物的抵抗^[67, 68]^。PD173074是一种选择性FGFR抑制剂，在体内及体外均可阻断SCLC的生长且当与顺铂联用时可显著改善疗效^[69]^。而且，在异种移植物中PD173074所诱导的完全缓解可持续6个月以上，这很可能是通过减少肿瘤内增殖并增加凋亡细胞死亡实现的^[69]^。

沙利度胺，一种谷氨酸衍生物，具有抗血管生成活性并可引起肿瘤中VEGF和bFGF的生成减少，在两项随机ⅡⅠ期临床试验中对沙利度胺进行了研究^[70, 71]^。在这两项研究中，化疗联合或不联合沙利度胺作为维持治疗，时间为2年。然而两项试验均未体现对预后的改善，且接受沙利度胺治疗的患者副反应更大^[70, 71]^。目前，FGF-R的小分子抑制剂，如XL228（亦可抑制IGF1-R、Src、Bcr-Abl），正处于早期临床试验阶段（www.clinicaltrials.gov）。

**血管内皮生长因子**。血管内皮生长因子（VEGF）家族由VEGF-A、VEGF-B、VEGF-C、VEGF-D和VEGF-E以及它们的三个VEGF受体（VEGFR1-3）所组成^[72]^。VEGF信号通路可使内皮细胞的增加增殖、迁移和侵袭性增强，从而介导肿瘤的血管生成^[72, 73]^。

据报道SCLC患者的VEGF水平较高，且与肿瘤分期、疾病进展、化疗耐药以及预后不良有关^[45]^。SCLC表达VEGFR1-3，且VEGFR-2与肿瘤生长和侵袭密切相关^[72]^。在SCLC中，VEGF/VEGFR自分泌信号通路介导增殖和转移，可被VEGFR-2及VEGFR-3的单克隆抗体抑制^[72]^。SU6668可抑制VEGFR、c-kit和FGF-R，在人肺肿瘤异体移植物中可阻断增殖和血管生成^[74]^。另一项临床前期试验证实在SCLC异种抑制模型中ZD6474（一种VEGFR-2和EGFR激酶抑制剂）可显著干扰VEGF信号和血管生成，并导致增殖减少和凋亡增加^[75]^。

抑制VEGF/VEGFR信号通路可能时一种有效的治疗策略。最近，两项针对广泛期SCLC患者的Ⅱ期临床试验取得了有利结论，这些患者均使用一线治疗联合贝伐珠单抗方案，并使用贝伐珠单抗作为维持治疗^[76, 77]^。一项评估贝伐珠单抗在广泛期SCLC中疗效的随机试验正在进行中（www.clinicaltrials.gov）。然而，在一项Ⅱ期临床试验中，范德替尼（ZD6474）作为维持治疗应用于已从一线化疗或放疗中获得最佳疗效的患者，结果显示患者无任何获益^[78]^。

## 细胞内分子伴侣

**热休克蛋白90**。热休克蛋白（HSP）-90是高度保守的分子伴侣家族成员之一，在新生肽链合成过程中行使新生蛋白（“宿主”）折叠的功能^[79]^。分子伴侣参与蛋白质构象成熟、蛋白质跨膜转位、内质网中蛋白质的质量控制以及正常的蛋白质循环^[80]^。分子伴侣在信号分子的翻译后调节、转录复合物的组合和分解^[81, 82]^以及免疫原性肽的加工处理中^[83, 84]^发挥作用。

Hsp90是一个组成性表达的细胞蛋白，在肿瘤细胞高应激状态下有所升高，这种高应激状态可由存在突变和失调蛋白、氧化损伤、缺氧或营养不良环境所引起^[85, 86]^。Hsp90的宿主蛋白包括许多癌基因蛋白，如AKT、MET、bcl-2、端粒酶、survivin和Apaf-1，通过持续的蛋白翻译和细胞增殖从而促进肿瘤细胞的生存、生长以及转移^[19, 86]^。因此，在恶性、转化细胞中^[85]^，抑制Hsp90通过降解癌基因并破坏多种信号途径可优先靶向作用于分子伴侣功能。

在SCLC中，存在抗凋亡蛋白的过表达及促凋亡分子的表达减少，从而破坏凋亡。在SCLC中Hsp90是主要的凋亡抑制因子^[19]^，这与其它的细胞系统不同。临床前试验数据表明在SCLC中抑制Hsp90可引起Apaf-1的释放并形成Apaf-1-caspase-9凋亡诱导复合物。只有被线粒体释放的细胞色素c激活后该复合物才能引起明显的凋亡，而细胞色素c的释放是由AKT失活和Hsp90抑制共同触发（图 2）。一旦AKT被降解，Bad将去磷酸化。Bad随即与抗凋亡Bcl-2家族成员形成异二聚体或激活促凋亡蛋白Bax和Bak，从而导致线粒体的去极化^[19, 87]^。因此，通过调控PI(3)K-AKT生存通路Hsp90作为Apaf-1的负性调控因子而调节凋亡^[19]^。

Hsp90抑制剂可引起Rb依赖的G_1_期停滞^[88]^。来自本研究机构的最新临床前期实验显示在含或不含Rb的细胞系中调控凋亡的机制存在一个有趣的差别。应用Hsp90抑制剂17-AAG处理后，大部分肿瘤细胞发生G_1_期阻滞，其原因可能是由于Rb介导的调控。然而，当缺乏Rb的细胞经17-AAG处理后，他们经过了G_1_期而停滞于M期，并在M期发生即时凋亡^[89]^。

许多Hsp90抑制剂已进入临床试验。首先研究的是格尔德霉素类似物、17-AAG和17-DMAG以及17-AAG删减后的衍生物IPI-504 ^[90, 91]^。正在早期试验阶段被评估的Hsp-90抑制剂的其它种类包括小分子嘌呤支架抑制剂^[90, 91]^（如CNF2024）、diarylpyrazole复合物（如3, 4 diarylisoxazole NVP-AUY922/VER-52296）以及有新的支架发展而来的SNX5422。

## 细胞表面标志物

**CD56（NCAM）**。神经细胞粘附分子（neural cell adhension molecular, NCAM）与免疫球蛋白家族相关，可调节神经内分泌细胞的生长、迁移和分化^[92]^。CD56是由NCAM基因编码的一个亚型。在几乎100%的SCLC中可见NCAM^[92]^。尽管其亦表达于自然杀伤细胞、神经内分泌腺体、中枢及周围神经系统以及心肌细胞中，它仍被作为抗癌治疗的一种靶点来进行研究^[92]^。恶性细胞受NCAM信号影响；NCAM的配体或抗体可发挥肿瘤进展抑制剂的作用^[92]^。BB10901是人源化鼠单克隆抗体N901通过二硫键与细胞毒性药物DM-1结合的免疫交联物，对CD56细胞系具有较强的选择性^[93]^。一项有关BB10901在复发性SCLC和CD56阳性小细胞癌患者中疗效的Ⅰ/Ⅱ期临床试验已经开展，且试验表明3/10的患者可观察到临床疗效且是安全的^[94]^。这项试验已接近于临床获益，但最终的结果仍悬而未决。

**神经节苷脂**。神经节苷脂是细胞膜组成成分的一种糖脂亚群，其在所有的真核细胞中尤其是在中枢神经系统中均可见^[95]^。据报道他们可作用于细胞膜受体和粘附分子^[95]^。在SCLC中可见这些抗原的表达增加^[95]^。Fucosyl GM-1可见于75%的SCLC标本中，而在正常组织或NSCLC及其它肿瘤中很少出现^[96]^。多聚涎酸为胚胎NCAM的一个组分，是一个超过20个负电荷通过alpha2-8连接的涎酸残基聚合物，其参与了细胞的运动和发育。在SCLC中大量表达，而在正常组织中则不表达^[97, 98]^。其它的几种神经节苷脂，如GM-2和Globo-H，可在多种肿瘤中表达。一种由fucosyl GM1、多聚涎酸、GM2和Globo H抗原所构成的四联疫苗已经被研发出来^[99]^。

## 进化通路

当被异常激活时，可调节干细胞自我更新的Hedgehop、Notch和Wnt等进化通路可引发肿瘤性增生，其意味着肿瘤发生的早期时间^[100-102]^。SCLC呈现典型的神经内分泌表型^[103]^，可表达神经内分泌标志物，如突触素、嗜铬素A和CD-56。在发育中的肺气道上皮细胞内，神经内分泌细胞是最早被识别的一种已分化的细胞类型^[104]^。这种由底层的内胚层分化成神经内分泌细胞的过程由Notch信号调控，而信号的异常可导致细胞腔隙的增大。有证据表明SCLC是分化最差的气道上皮肿瘤，非常接近与早期发育中的肺组织^[105]^。SCLC依赖变异的Notch信号以及Hedgehog信号的激活，这均类似于早期的肺形成过程（见图 4）^[106]^。针对这些通路的靶向治疗可能会清除SCLC的克隆原细胞，并获得更持久的治疗效果。

**4:**
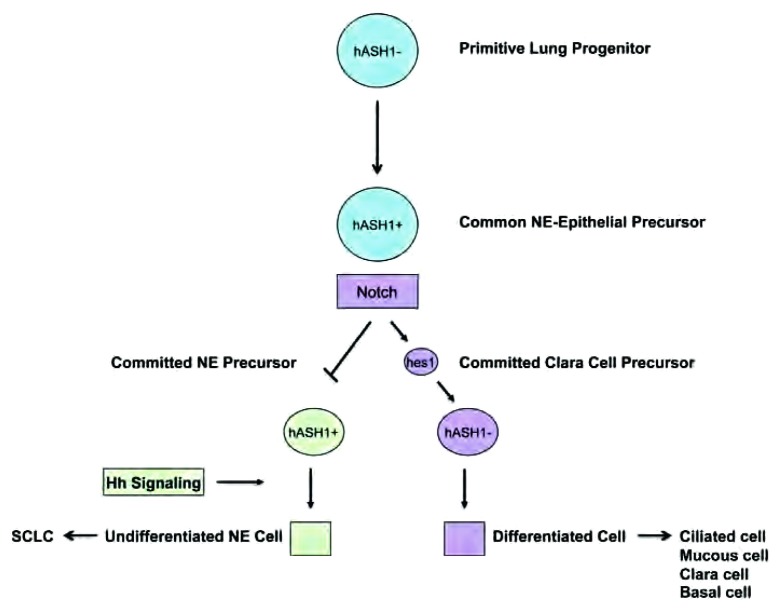
SCLC呈典型的神经内分泌表型，其依赖于Notch信号的异常以及Hedgehog信号的激活，这与早期肺脏的形成过程相似。NE细胞是在发育中的肺气道上皮中最早被鉴定出的分化型细胞类型。而h-ASH-1是神经内分泌细胞发育所必须的。Notch信号通路的增加可活化转录靶点，包括阻滞h-ASH-1转录的Hes1。当细胞h-ASH-1表达缺乏时，它们被固定地分化成特定细胞系。因此，增加Notch信号通路可造成SCLC的生长抑制。在这些上皮内细胞群中，Hedgehog信号通路恰好在罕见未分化神经内分泌细胞发育之前，失调增加可导致SCLC。引自Ball DW^[119]^。 SCLC, which exhibits a characteristic neuroendocrine (NE) phenotype, depends on aberrations in Notch signaling and on activation of Hedgehog signaling similar to early lung formation. NE cells are the first identifiable differentiated cell type in airway epithelium in the developing lung. Human achaete-scute homolog-1 (h-ASH-1) is required for the development of neuroendocrine cells. Increased Notch signaling leads to activation of transcriptional targets, including Hairy enhancer of split 1 (Hes1), which blocks the transcription of h-ASH-1. When cells lack expression of h-ASH-1, they become committed to a differentiated cell line. Thus, increased Notch signaling can cause growth inhibition of SCLC. Hedgehog signaling in this intraepithelial cell population directly precedes the development of the rarer undifferentiated neuroendocrine cells, and increased dysregulation leads to SCLC. Adopted from Ball DW.

**Hedgehog通路**。研究表明Hedgehog通路（Hh）在早期肺的形成和发育过程中非常重要，其可调节上皮-间质的相互作用^[100, 107]^。在人类中这条通路存在三种已知的配体：Sonic Hedgehog（SHh）、Indian Hedgehog（Ihh）以及Desert Hedgehog（DHh）。信号级联的启动是由Hh与Patched-1受体的结合触发的（Ptch-1），Ptch-1是一种12跨膜蛋白。在Hh配体缺失的情况下，Ptch-1可结构性抑制7跨膜Smoothened（Smo），并使通道失活。然而，Hh配体与Ptch-1的结合可使受抑制的Smo被释放，可激活一种蛋白复合物，而Hh的下游转录因子可靶向作用于包括Gli-1和Ptch-1在内的胞核。尽管在成人支气管上皮细胞基底层内呈现较低水平，但活化的Hedgehog信号通路可在由萘损伤所致的气道再生过程中引起上皮内细胞群的扩张^[107]^。上皮内细胞群中，Hedgehog信号通路的活化恰好在罕见气道神经内分泌细胞的发育之前^[107]^。在SCLC中，存在配体依赖的邻分泌形式的Hedgehog通路的激活，而邻近的细胞表达SHh^[100, 106, 107]^。此外，体外及体内试验表明，SCLC可被甾体类生物碱Hedgehog拮抗剂环巴胺（cyclopamine）抑制^[106, 107]^。这些数据支持在SCLC中存在祖细胞，其可维持化疗耐药并依赖可作为靶点的Hedgehog的进化通路。

Hedgehog抑制剂正处于临床试验阶段。Smo的直接抑制剂包括一种环巴胺类似物IPI-926（Infinity Pharmaceuticals, Inc., ）以及LDE225（Novartis Oncology），两者均处于Ⅰ期实验阶段。GDC-0449，也是一种Smo抑制剂，目前正处于ECOG 1508评估阶段，ECOG 1508为一项关于广泛期SCLC患者的随机Ⅱ期临床试验。另外两个正在进行Ⅰ期临床试验阶段的Hedgehog抑制剂是XL139（Bristol-Myers Squibb Conpany and Exelixis, Inc.）和PF-04449913（Pfizer）。

**Notch信号通路**。在不同背景下Notch信号通路可调节化、发育和细胞命运^[4, 108]^。更为重要的是，Notch通路在发育中的及成人组织中具有保护未成型及多潜能细胞的功能^[109, 110]^。在哺乳动物中，Notch通路具有4种跨膜Notch受体（Notch1-4）^[108]^，它们可被邻接细胞的3种Notch配体（Delta 1、Jagged 1和Jagged 2）所激活^[100]^。Notch通路对于调节气道上皮的发育非常关键，特别是可决定细胞神经内分泌或非神经内分泌的分化^[104]^。简单地说，Notch信号通路可导致转录靶点的激活，如发状分裂相关增强子-1（hairy enhancer of split 1, Hes1）^[111]^。反过来，Hes-1可阻断肺神经内分泌细胞发育所必须的h-ASH-1^[104]^。*Hes*-1敲除的小鼠的肺中，鼠ash-1表达上调，神经内分泌细胞增加，克拉拉细胞（Clara cell）相应减少^[112]^。在SCLC中，h-ASH-1高表达，而Notch-1则是失活的^[104]^。Notch受体的过表达可导致细胞周期停滞及SCLC的生长抑制^[103]^。因此，激活Notch-1信号通路可能是一项有效的治疗SCLC的策略。

**WNT**。Wnt蛋白由包括19个分泌型分子家族组成，具有不同的表达模式和功能，包括增殖、分化、存活、凋亡和细胞运动^[113]^。Wnt信号通过以下三个途径之一发挥作用。首先是“经典的”信号通路，配体与卷轴受体（Fz受体）及LDL受体相关蛋白（LRP）相结合，维持胞浆的稳定以及靶基因表达（Wnt/β-catenin通路）所需β-catenin的核内转运^[114]^。第二个信号通路是通过钙调蛋白激酶Ⅱ和蛋白激酶C（Wnt/Ca^2+^通路）的激活完成，可平衡经典途径^[115]^。第三，平面细胞极性通路，通过小GTPases而起作用，如RhoA和Jun激酶（JNK），与细胞骨架重排和细胞的极性有关^[115]^。

在肺的形态发生过程中，特定的Wnt信号对于正常的上皮-间质相互作用是必需的。当Wnt信号通路失调时，有害事件便会发生^[116]^。在成人的肺中，Wnt信号通路的所有组分均维持在可检测水平。支气管肺泡干细胞可共同表达克拉拉及上皮细胞标记蛋白，可由Wnt信号维持和活化^[116]^。当支气管细胞暴露于烟草烟雾中时，Wnt信号通路被激活，从而导致增殖和肿瘤生长^[117]^。在NSCLC标本中，Wnt分子差异表达，Wnt蛋白上调（如，Wnt1和Wnt 2），而Wnt调节因子的表达减少（如WIF）^[116]^。靶向作用于这条通路可能是肿瘤控制的有效途径。研究表明许多方法在NSCLC中有效，如阻断卷轴受体、通过siRNA或单克隆抗体抑制Wnt^[118]^。然而无一得到临床验证。

## 结论

如本文所述，在SCLC的发病机制有许多不同的途径，这导致其具有独特的生物学和临床特征。因此，理解其基本的分子及细胞学改变有利于研发新的治疗策略提供坚实的基础。为最终改善SCLC患者的生存时间，众多的分子靶向治疗药物正处于临床及临床前期研究中。

## Acknowledgements

The authors would like to acknowledge Dr. Lee M. Krug for his thoughtful review of the manuscript.
